# Interactions between Neighborhood Social Environment and Walkability to Explain Belgian Older Adults’ Physical Activity and Sedentary Time

**DOI:** 10.3390/ijerph13060569

**Published:** 2016-06-07

**Authors:** Veerle Van Holle, Jelle Van Cauwenberg, Ilse De Bourdeaudhuij, Benedicte Deforche, Nico Van de Weghe, Delfien Van Dyck

**Affiliations:** 1 Department of Movement and Sports Sciences, Ghent University, Watersportlaan 2, Ghent B-9000, Belgium; veerle.vanholle@ugent.be (V.V.H.); ilse.debourdeaudhuij@ugent.be (I.D.B.); 2 Research Foundation Flanders (FWO), Egmontstraat 5, Brussels B-1000, Belgium; jelle.vancauwenberg@ugent.be; 3 Department of Public Health, Ghent University, De Pintelaan 185, 4K3, Ghent B-9000, Belgium; benedicte.deforche@ugent.be; 4 Department of Human Biometry and Biomechanics, Vrije Universiteit Brussel, Pleinlaan 2, Brussels B-1050, Belgium; 5 Department of Geography, Ghent University, Krijgslaan 281 (S8), Ghent B-9000, Belgium; nico.vandeweghe@ugent.be

**Keywords:** aging in place, elderly, walking, sitting, socio-ecological models

## Abstract

This study examined associations between neighborhood social factors and physical activity (PA) and sedentary behavior (SB) in older adults. Furthermore, possible moderating effects of neighborhood walkability were explored. Data from 431 community-dwelling Belgian older adults (≥65 years) were analyzed. Neighborhood social factors included measures of neighboring, social trust and cohesion and social diversity. Neighborhood walkability was measured objectively. Outcome measures were self-reported weekly minutes of domain-specific walking and TV viewing, and accelerometer-assessed weekly minutes of moderate-to-vigorous physical activity (MVPA) and overall SB. A higher frequency of talking to neighbors was associated with higher levels of self-reported walking for transport and for recreation. Moderation analyses showed that only in highly-walkable neighborhoods, higher social diversity of the neighborhood environment was associated with more transport walking; and talking to neighbors and social interactions among neighbors were negatively associated with overall SB and television viewing, respectively. Findings suggest that a combination of a favorable neighborhood social and physical environment are important to promote older adults’ PA and limit SB.

## 1. Introduction

Regularly engaging in moderate-to-vigorous physical activity (MVPA) leads to several health benefits, also in older adults [1]. Physical activity (PA) slows down the progression of functional decline [2,3] and contributes to a reduction of the risk for adverse effects on function- and disability-related outcomes [4]. In addition, an emerging body of research suggests detrimental health effects of high levels of sedentary behavior (SB) (e.g., higher risk for metabolic syndrome, less favorable levels of cardio metabolic biomarkers [5]), which may even occur independently of whether or not people are sufficiently active [6,7]. Some studies have shown that with aging, the volume of PA tends to decrease [8] whereas the volume of SB expands [9], making older adults (≥65 years) the least physically active and most sedentary amongst all age groups [10,11,12]. Hence, due to aging-related behavioral and physiological changes (e.g., sarcopenia [13]) older adults are at a higher risk for adverse health effects and, eventually, institutionalization.

A clear understanding of the correlates and determinants of older adults’ PA and SB is needed before effective promotion strategies targeting higher PA and less SB can be created [14]. Socio-ecological frameworks posit that both behaviors are influenced by an interaction between an individual and its environment [15]. The neighborhood, including both social and physical environmental characteristics, may be an important behavior setting in older adults, as due to retirement, a significant part of their day is spent in the vicinity of the home residence.

The neighborhood social environment comprises different components [16], of which social capital is most widely investigated. Putnam [17] described it as ‘features of social organization such as networks, norms, and social trust that facilitate coordination and cooperation for mutual benefit’. Subcomponents include social participation, social trust, neighboring and neighborhood cohesion [18]. Cohesion [19,20], social participation [21] and social contacts [21,22] have been previously related to higher levels of mid-aged and older adults’ PA. Emerging research also observed relationships between higher levels of volunteering and participation and lower levels of television (TV) viewing in this age group [23]. In contrast, some studies could not identify associations between the social environment (e.g., social cohesion) and mid-aged or older adults’ PA [22,24]. Next to social capital, other potential social environment correlates of health behaviors include neighborhood social composition [25]. This factor has been associated with more transport walking [21] among older adults, however not to lower levels of TV viewing [23]. Overall, studies on social environmental correlates of older adults’ PA and SB are scarce [26].

Regarding the neighborhood physical environment, a commonly studied feature is walkability, reflecting the environment’s convenience for PA, primarily transport walking [27]. Recent literature consistently showed that walkability is positively related to older adults’ PA [28,29] and it may also associate with lower levels of their SB [30].

Socio-ecological models emphasize the importance of examining interactions between different levels of health behavior correlates [15]. Such interactions are also illustrated in Alfonzo’s Hierarchy of Walking Needs [31], which classifies environmental factors into hierarchical categories or ‘needs’ and posits that lower-order needs can only enhance walking if higher-order needs are fulfilled. Given that the highest-order need in the Hierarchy is the environment’s accessibility [31], it can be expected that the social environment is more convenient towards higher levels of PA in a high-walkable environment. Analogously, this may also hold true with regard to lowering SB levels. Today, to our knowledge, no studies have addressed interactions between neighborhood social and physical environmental factors to explain PA levels [16] or SB in older adults [26].

The aims of the present study were twofold. Firstly, we examined main associations between the neighborhood social environment and older adults’ PA and SB. It was hypothesized that higher social environment scores would relate to higher levels of PA and lower levels of SB. Secondly, we investigated moderating effects of objectively-measured neighborhood walkability. We hypothesized that the potential association between the neighborhood social environment and PA/SB would be more pronounced in highly-walkable neighborhoods.

## 2. Materials and Methods

### 2.1. Sampling and Procedures

Cross-sectional data from BEPAS Seniors were used. Data were collected between October 2010 and September 2012 among community-dwelling older adults (≥65 years) living in 20 neighborhoods located in Ghent (Belgium). A detailed description of the neighborhood selection procedure and participant recruitment can be retrieved elsewhere [29].

In total, 508 community-dwelling older adults participated (response rate 44.8%). Responders gave written consent and were visited twice by a trained interviewer, and wore an ActiGraph GT3X(+) accelerometer in between two visits for seven consecutive days. The first visit assessed self-reported physical functioning, PA and TV-viewing. The second home visit consisted of collecting the accelerometer and an interview assessing socio-demographics and the neighborhood social environment. The study protocol was approved by the Ethics Committee of the Ghent University Hospital (Belgian registration number B670201423000). 

### 2.2. Measures

#### 2.2.1. Independent Variables

##### The Neighborhood Social Environment

The neighborhood social environment variables represent measures of neighborhood social capital components (neighboring variables and social trust and cohesion), and neighborhood social diversity. Details on all variables’ content can be retrieved in Supplementary Material Table S1.

Two variables measured neighboring activities, *i.e.*, the degree of casual social interactions between older adults and their neighbors [18,32]. ‘Talking to neighbors’ assessed the frequency of participants’ informal social interactions with neighbors. ‘Social interactions among neighbors’ represented formal social interactions. ’Neighborhood social trust and cohesion’ was calculated as participants’ agreement with statements about their local neighborhood [33]. Furthermore, ‘neighborhood social diversity’ was calculated analogous to previous research in older adults [21] as an average measure of three items related to the social composition of the neighborhood.

##### Physical Environment: GIS-Based Neighborhood Walkability

Geographic Information System (GIS) data on residential density, street connectivity and land use mix diversity were used to calculate a neighborhood-level walkability index, adapted from Frank *et al.* [27]: Walkability = (2 × z-connectivity) + (z-residential density) + (z-land use mix) [34]. Neighborhoods in the top and bottom walkability quartiles were designated as representing high and low walkability, respectively.

#### 2.2.2. Outcome Measures

Since different correlates may be related to different sub-behaviors of PA [35,36] and SB [37,38], domain-specific (self-reported), as well as overall (accelerometer-based) measures of PA and SB were investigated.

##### Self-Reported PA and SB

Self-reported PA levels were assessed using an elderly-adapted Belgian version of the interviewer-administered long International Physical Activity Questionnaire [39]) [40]. Participants reported the time they spent doing work-related, domestic, transport-related and recreational activities during the previous seven days; each with a minimum duration of 10 min. Only self-reported transport and recreational walking (min/week) were retained as outcome measures, because walking is the most popular type of older adults’ PA [41,42].

Weekly minutes of TV viewing time was assessed through an item with the same format as the IPAQ, which has been shown acceptably reliable [43]. Data on total self-reported PA or SB (calculated as a summary score of all reported domain-specific PA/SB, expressed in minutes per week) were used for preliminary screening on outliers (*i.e.*, self-reports of >6720 min/week or 16 h/day PA were excluded [44] (*n* = 21)).

##### Accelerometer-Based PA and SB

Overall MVPA and SB were assessed with the valid and reliable GT3X(+) hip-mounted accelerometers (ActiGraph, Fort Walton Beach, FL, USA) [45,46,47], which were worn during waking hours on seven consecutive days. Data capturing the vertical plane were collected using 60-s epochs [48]. Subsequent to downloading raw data, accelerometer files were screened and scored using MeterPlus 4.3 (Santech, Inc., San Diego, CA, USA; www.meterplussoftware.com). Twenty-eight participants were excluded because of accelerometer device failure. For the remaining files, a valid day was defined as a minimum of 10 wearing hours and participants with <five valid days of data were excluded [48] (*n* = 22). Participants with registrations over 18 daily hours were also excluded [49] (*n* = 6) due to likelihood of wearing beyond waking hours as per study protocol. Apart from “non-wearing” (≥90 min of consecutive zeros [50]), scored categories were “sedentary” (registrations <101 counts·min^−1^) and “MVPA” (registrations ≥1952 counts·min^−1^ [51], which were converted into weekly minutes of total SB and MVPA, respectively.

#### 2.2.3. Covariates

Participants self-reported their age, current living situation, educational attainment, former occupational status, and number of cars/motorized vehicles in the household. Self-reported physical functioning was measured with the Short Form 36 item Survey (SF-36; [52]). Activities (*n* = 10) in which participants reported to be severely/somewhat limited were summed and this measure was dichotomized at its median value (2 limitations). Residential self-selection was measured using an eight-item scale [49] adapted from Frank *et al.* [53]. Lastly, neighborhood income (annual household median; high *vs.* low) was included as a covariate.

### 2.3. Statistical Analyses

Full data from 431 older adults were included All outcome variables were square root transformed to improve normality in the data. Except for descriptive statistics, which were calculated with the raw data in SPSS 22.0, analyses reported below used square root transformed variables.

Multilevel (neighborhood-participant) linear regression models were conducted in MLwiN 2.30 to determine associations between neighborhood social environmental factors and all outcome variables, and moderating effects of neighborhood walkability. Firstly, single models (main and interaction effects) were ran for each social environmental and outcome variable separately, these can be retrieved in Supplementary Material Tables S2 and S3. Secondly, a multivariable model was built, including all terms yielding *p* < 0.10 in the first step. For these multivariable models, statistical significance was set at *p* < 0.05 for interpreting main effects and at *p* < 0.10 for interpreting moderating effects [54].

All analyses were adjusted for gender, educational attainment, living situation, age, residential self-selection, car ownership and neighborhood income. Additionally, analyses for TV viewing and overall objective SB were respectively adjusted for self-reported leisure-time PA (sum of recreational walking, cycling and other recreational MVPA) and objectively-measured MVPA. Analyses for accelerometer-based MVPA and SB were adjusted for number of accelerometer wearing days and valid wearing hours. Lastly, as in previous analyses [29], walkability was only related to MVPA in low-income neighborhoods, three-way interactions between walkability, neighborhood income and the social environmental variables were examined for this outcome measure.

## 3. Results

### 3.1. Sample Characteristics

Table 1 displays socio-demographic and behavioral characteristics of the sample, as well as descriptive statistics of the neighborhood social environment variables. In total 431 Belgian older adults (54.5% females) with a mean age of 74.4 ± 6.2 years were included for analysis.

### 3.2. The Neighborhood Social Environment and PA, and Moderation Effects

Multivariable results for PA outcome measures can be retrieved in Table 2 (MVPA) and Table 3 (walking). Frequency of talking to neighbors was positively associated with higher self-reported transport (*p* = 0.002) and recreational walking (*p* = 0.004). Compared to their peers with the lowest frequencies of talking to neighbors, older adults with the highest frequencies of talking to neighbors reported an additional 42.5 and 41.5 weekly minutes of transport and recreational walking, respectively. No main associations of social interactions among neighbors, social trust and cohesion, or the neighborhood social diversity with the three PA outcome measures were observed.

A three-way interaction between walkability, neighborhood income and social trust and cohesion was observed for MVPA. In low-income, high-walkability neighborhoods, there was a negative association between social trust and cohesion and older adults’ MVPA engagement (*p* = 0.081). Residents of these neighborhoods who perceived the highest levels of social trust and cohesion, accumulated 92.3 fewer weekly minutes of MVPA than their counterparts with the poorest levels of social trust and cohesion. In the other types of neighborhoods, social trust and cohesion was not associated with older adults’ MVPA.

**Figure 1:**
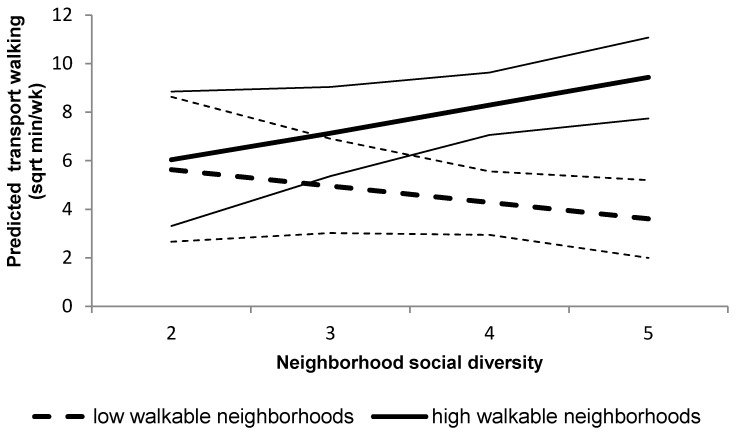
Interaction between the neighborhood social diversity and neighborhood walkability for the predicted transport walking. Plot represents transport walking (square root transformed variable) for high-walkability (thicker full line) and low-walkability (thicker dashed line) neighborhood residents, and their confidence intervals (thinner lines).

Walkability moderated the association between neighborhood social diversity and transport walking (*p* = 0.036; see Figure 1), showing that residents who perceived a more diverse social composition of the neighborhood, reported higher levels of transport walking if they also resided in a high-walkable neighborhood (*p* = 0.056). Those older adults who perceived the social composition to be most diverse, reported an additional 56.5 weekly minutes of transport walking, compared to older adults who reported the least diverse neighborhood social composition. In low-walkable neighborhoods, neighborhood social diversity was not related to transport walking (*p* = 0.289). No other interactions between the neighborhood social environment and walkability were observed for the walking outcomes.

### 3.3. The Neighborhood Social Environment and SB, and Moderation Effects

Findings regarding the SB outcome measures can be found in Table 3. No multivariable main associations with the two SB outcome variables were retrieved.

**Figure 2:**
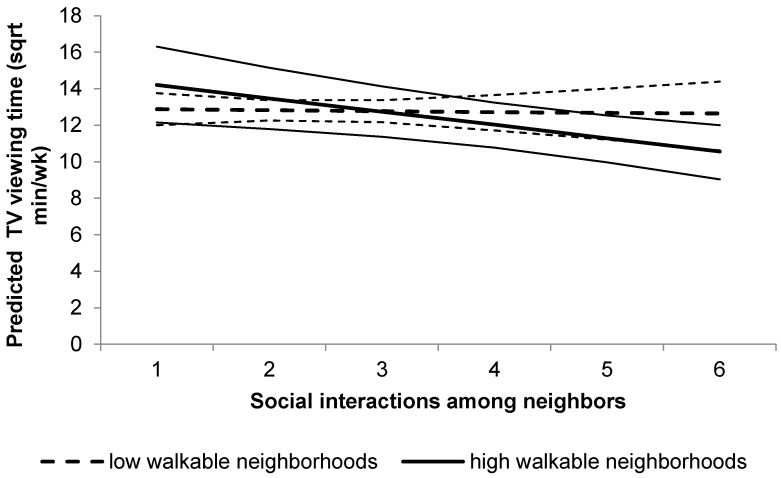
Interaction between social interactions among neighbors and neighborhood walkability for the predicted TV viewing time. Plot represents TV viewing levels (square root transformed variable) for high-walkability (thicker full line) and low-walkability (thicker dashed line) neighborhood residents, and their confidence intervals (thinner lines).

Social interactions among neighbors interacted with walkability for TV viewing (*p* = 0.059): a higher frequency of social interactions among neighbors was associated with less TV viewing time if older adults also resided in highly-walkable neighborhoods (*p* = 0.004), with 94.5 fewer weekly minutes of self-reported TV viewing time for those older adults with the highest level of social interactions, compared to their peers with the lowest level. In contrast, no association between social interactions and TV viewing time was observed in low-walkable neighborhoods (*p* = 0.827; see Figure 2). 

**Figure 3:**
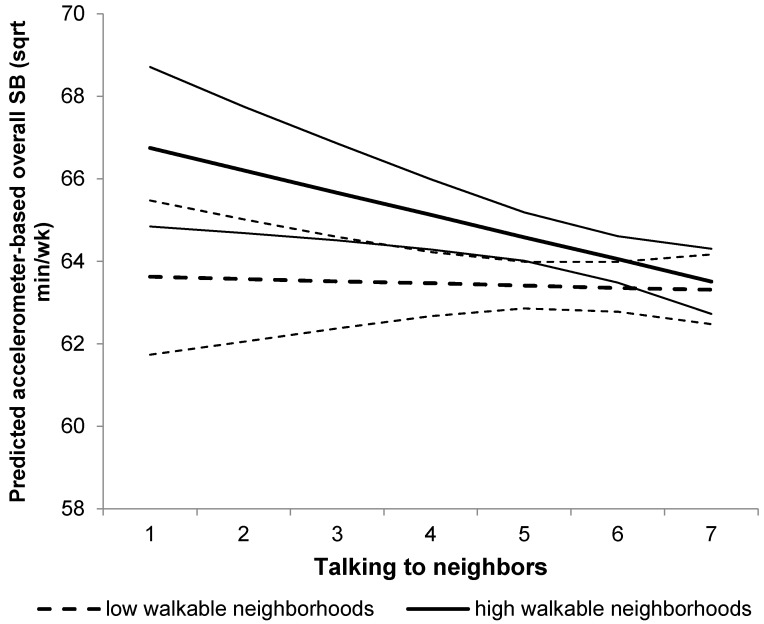
Interaction between talking to neighbors and neighborhood walkability for the predicted overall SB. Plot represents overall SB levels (square root transformed variable) for high-walkability (thicker full line) and low-walkability (thicker dashed line) neighborhood residents, and their confidence intervals (thinner lines).

For accelerometer-based total SB, a significant interaction between walkability and talking to neighbors was observed (*p* = 0.081; Figure 3). Higher frequencies of talking to neighbors were related to lower levels of overall sitting time (a difference of 421.5 fewer weekly minutes between older adults with the highest *versus* lowest frequency of talking to neighbors), in highly-walkable (*p* = 0.020), but not low-walkable neighborhoods (*p* = 0.825). No other interactions between neighborhood social environmental factors and walkability were observed for the SB outcome variables.

## 4. Discussion

The present study observed positive main associations between frequencies of talking to neighbors and older adults’ walking, but not with SB outcomes. Interactions between the social environment factors and neighborhood walkability revealed that in residents of highly-walkable neighborhoods, higher scores on social environmental factors were related to higher walking for transport, less TV viewing and less overall SB.

Both in high- and low-walkability neighborhoods, higher frequencies of talking to neighbors were associated with more transport and recreational walking, complementing previous research reporting positive associations between frequency of neighborhood contacts and transport walking [21]. This finding suggests that the frequency of informal social contacts may be important to invite older adults to get outdoors in their residential neighborhood and consequently stimulate them to walk [55], which is in accordance with qualitative research reporting that older adults mentioned the opportunity for social interaction as an important reason to engage in transport walking [56,57] and other physical activities [58]. Otherwise, since our results are cross-sectional, it is also possible that the observed association is reciprocal and older adults who engage in higher levels of walking are more likely to have incidental social encounters with neighbors [59]. Informal socialization has been mentioned by older adults as a facilitator of PA events themselves [57] and this might also suggest that higher levels of PA could enhance the social well-being of older adults [60].

However, no direct associations with PA or SB were observed for the other neighboring variable, social interactions among neighbors, reflecting the frequency of older adults’ formal social contacts with neighbors. This contrasts with other research on older adults describing positive associations between neighbors’ formal social support and walking [21,57], but is in line with previous work, where neighborhood contacts were not related to older adults’ TV viewing time [23]. More research on the possible association between neighboring and older adults’ PA and SB is recommended to reveal consistent relations.

Although no direct associations between social environment factors and SB’s were observed, interaction analyses identified that walkability moderated the association between talking to neighbors and overall SB, as well as the relationship between social interactions among neighbors and TV viewing, and the associations between perceptions of social diversity in the neighborhood and more transport walking. All interactions indicated that only in highly-walkable neighborhoods, higher scores on the social environment variables were related to less overall SB, less TV viewing and more transport walking. For transport walking and TV viewing time, the observed walkability × social environment interactions also showed synergistic effects of combining both a supportive social and physical environment. Such synergy is one of the core principles of socio-ecological models [15]. The observed interactions in the present study are discussed in further detail below. To the best our knowledge, no previous studies examined interactions between neighborhood walkability and social environment factors in relation to older adults’ PA or SB. Hence, our results cannot be compared to existing research, but some possible explanations for our findings are provided.

It was shown that perceiving a higher social diversity in the neighborhood was a positive correlate of transport walking in older adults living in highly-walkable neighborhoods. In a previous study, it was shown that older adults living in highly-walkable neighborhoods, walk more for transport [29] and perceiving a diverse social composition seemed to strengthen this relationship.

It could be possible that the social composition of the neighborhood might be more proximal to health behavior in highly-walkable neighborhoods. Potentially, in such neighborhoods, social composition might be related to lower perceived crime [61], which has, to some extent, been associated to higher engagement in PA [62]. In low-walkability neighborhoods, transport walking may already be less common because of the physical environmental design (e.g., fewer destinations nearby) [29] and this may be the convincing factor for not engaging in transport walking, irrespective of the neighborhood social composition. If destinations are not within a walkable distance, other factors may play less of a role in inviting older adults to walk for transport [63].

With regard to the interactions in relation to overall SB, results suggested that enhancing the opportunities for informal social contacts with neighbors alone might not be sufficient to reduce older adults’ SB, whereas the combination with living in a walkable neighborhood may be. Furthermore, in highly-walkable neighborhoods, older adults reported less TV viewing time when they had higher levels of formal social interactions among neighbors. Those high-walkability neighborhood residents with broader social networks in the neighborhood could have replaced their indoor TV viewing by more active alternatives, such as engagement in light or moderate PA during social activities with their neighbors. In highly-walkable neighborhoods, it may be more likely that social activities with neighbors involve activities outside the house, because they have sufficient significant destinations that can serve as alternatives for replacing their TV viewing. For instance, access to alternative activities and presence of cultural facilities were related to less TV viewing in another Belgian study [23]. In contrast, low-walkable neighborhood residents have fewer destinations nearby [27], and therefore, activities with their neighbors may more often involve indoors sedentary activities including TV viewing.

It should be noted that regarding social trust and cohesion, we only observed that in low-income, high-walkability neighborhoods, poorer levels of social trust and cohesion were related to higher levels of MVPA. A clear explanation for this observation is hard to formulate. For this type of neighborhoods, our finding contrasts with some previous results in mid-aged and older adults, where social cohesion was positively related to higher levels of PA [19,20,64]. Moreover, Mollenkopf *et al.* [65] demonstrated that those older adults who were more socially tied, were also the ones who got out of the house more often. Nonetheless, the lack of association between social trust and cohesion and all other outcome measures (*i.e.*, transport and recreational walking, TV viewing and accelerometer SB) confirms other studies in mid-aged or older adults [22,24]. Anyhow, more research is warranted on the role of social trust and cohesion in explaining Western-European older adults’ PA and SB behaviors.

### Strengths and Limitations

Strengths of the present study include that we obtained both self-reported and objective outcome measures. The self-reported levels of walking and TV viewing were assessed through face-to-face interviews. It has been shown that in adults, reporting bias was lower when an interviewer-administered version *versus* a self-administered version was used to assess health behaviors such as PA [66]. Interviewer guidance is likely to be even more beneficial in older adults, because this age group may experience more cognitive difficulties when responding to a questionnaire [67,68]. Nevertheless, it is also important to have objective measures of overall MVPA and SB, since overall MVPA levels are likely to be over-reported [40] and overall SB is likely to be underreported [42] by older adults. However, a limitation of accelerometer assessment in this sample includes that the applied cut points of Freedson *et al.* [51] were calibrated in younger adults, which may not be directly transferable to older-aged populations because of differences in resting metabolic rate between younger and older adults [69]. Furthermore, the study was limited by its cross-sectional design and consequently, causality of the findings could not be established. For instance, it might be that older adults who are more active, have more neighborhood (in)formal social interactions through their activities. Hence, the associations and interactions observed in the present study should be carefully interpreted. Secondly, generalizability of the present study findings may be limited. Compared to the Belgian population of older adults [70], a greater proportion of older adults in the present study reported to have a higher educational degree and a higher proportion lived with a partner.

## 5. Conclusions

To conclude, the current study findings suggested direct associations between the neighborhood social environment and older adults’ walking, but not SB. Since only the talking to neighbors variable was a significant correlate of walking, this might show that brief interactions in this age group could be important in relation to being active or not. Nonetheless, the findings on possible interactions demonstrated that, in highly-walkable neighborhoods, social diversity was related to higher walking levels and higher neighboring scores were related to lower SB levels. Analogous to Alfonzo’s Hierarchy of Walking Needs [31], our findings stress that if interventions would aim at increasing older adults’ PA and lowering their SB, creating neighborhoods with a high accessibility of significant destinations may be a necessary prerequisite. The presence of significant neighborhood destinations where older adults can engage in social interactions may be key.

## Figures and Tables

**Table 1:** Socio-demographics, neighborhood social environment and older adults’ PA and SB levels.

Socio-Demographics	*N* = 431	
Gender (% female)	54.5	
Age in years (M ± SD)	74.4 ± 6.2	
Living situation (% with partner)	65.5	
Educational level (% tertiary)	38.3	
Former occupation (%)		
household	18.5	
blue collar	26.4	
white collar	55.1	
**Neighborhood Social Environment**	**M ± SD**	**Range**
Talking to neighbors ^1^ (/7)	5.5 ± 1.4	1.0–7.0
Social interactions among neighbors ^1^ (/7)	2.2 ± 1.1	1.0–6.3
Social trust and cohesion neighborhood ^1^ (/5)	3.7 ± 0.8	1.0–5.0
Social diversity neighborhood ^2^ (/5)	4.2 ± 0.7	1.7–5.0
**Dependent variables**	**M ± SD**	**MED; IQR**
Transport walking^3^ (min·week^−1^)	86.8 ± 141.9	30.0; 0.0–120.0
Recreational walking^3^ (min·week^−1^)	83.2 ± 159.3	0.0; 0.0–120.0
TV^3^ (min·week^−1^)	1191.4 ± 738.5	1260.0; 630.0–1680.0
MVPA^4^ (min·week^−1^)	111.7 ± 117.2	72.0; 23.3–165.0
Sedentary behavior ^4^ (min·week^−1^)	4039.1 ± 708.0	4109.5; 3581.3–4565.3

Numbers represent mean ± standard deviations, unless indicated otherwise; PA = physical activity; MVPA = moderate-to-vigorous PA; SD = standard deviation; IQR = interquartile range; ^1^ higher scores represent higher frequency of talking to neighbors or social interactions, or higher trust and cohesion; ^2^ higher scores represent higher social diversity; ^3^ self-reported; ^4^ accelerometer-based.

**Table 2:** Multivariable model for associations between social environment factors and MVPA, and interactions with neighborhood walkability and income.

Social Environment Factor	Main Effect Walkability	Main Effect Income (Ref. = Low)	Main Effect Soc. Env. Factor	Income × Walkability	Income × Soc. Env. Factor	Walkability × Soc. Env. Factor	Walkability × Income × Soc. Env. Factor
	**B ± SE**	**B ± SE**	**B ± SE**	**B ± SE**	**B ± SE**	**B ± SE**	**B ± SE**
	2.31 ± 1.70	0.43 ± 0.75		**−1.82 ± 1.08 ^¥^**			
Interactions neighbors			0.20 ± 0.46		−0.65 ± 0.62	−0.18 ± 0.68	0.96 ± 0.92
Social trust &cohesion			0.67 ± 0.61		−0.24 ± 0.89	−1.71 ± 0.85 *	**2.20 ± 1.21 ^¥^**

B = regression coefficient; SE = standard error; * *p* < 0.05; **^¥^** *p* < 0.10. The outcome variable (MVPA) was square root transformed; main effects, two-way and three-way interactions were calculated for each social environmental factor, adj. for number of valid accelerometer wearing days, number of accelerometer hours on valid days, gender, age, living situation, residential self-selection, car ownership and educational attainment.

**Table 3:** Multivariable model for main associations between social environment factors and walking/SB, and moderating effects of walkability.

Independent Variables	Walking Transport ^1^	Walking Recreation ^1^	Overall SB ^2^	TV Viewing ^3^
	Multivariable Model	Multivariable Model	Multivariable Model	Multivariable Model
	B ± SE	B ± SE	B ± SE	B ± SE
**Main effects**				
Talking to neighbors	0.68 ± 0.23 *	0.80 ± 0.27 *		
Neighborhood social diversity		0.98 ± 0.54 ^¥^		−0.44 ± 0.28
**Interactions**				
Walkability × talking to neighbors			−0.56 ± 0.30 ^¥^	
Walkability × social interactions among neighbors				−0.65 ± 0.36 ^¥^
Walkability × social diversity	2.31 ± 0.90 *			

B = regression coefficient; SE = standard error; * *p* < 0.05; ^¥^ *p* < 0.10. All outcome variables were square root transformed; ^1^ adjusted for gender, age, living situation, residential self-selection, motorized vehicle ownership and educational attainment; ^2^ adjusted for number of accelerometer wearing days, number of accelerometer wearing hours per valid day, MVPA, gender, age, living situation, residential self-selection, motorized vehicle ownership and educational attainment; ^3^ adjusted for leisure-time PA, gender, age, living situation, residential self-selection, motorized vehicle ownership and educational attainment.

## Supplementary Materials

The following are available online at www.mdpi.com/1660-4601/13/6/569/s1, Table S1. Content and scoring of neighborhood social environment variables. Table S2. Associations between social environment factors and MVPA, and interactions with neighborhood walkability and income (single models). Table S3. Single models for main associations between social environment factors and walking/SB, and moderating effects of walkability.

## Abbreviations

The following abbreviations are used in this manuscript:

PA	physical activity
MVPA	moderate-to-vigorous physical activity
SB	Sedentary behavior
GIS	Geographic Information Systems
