# Supplementary material for: Interactions between Neighborhood Social Environment and Walkability to Explain Belgian Older Adults’ Physical Activity and Sedentary Time
Source: Int J Environ Res Public Health. 2016 Jun 7;13(6):569. doi: 10.3390/ijerph13060569 (PMC4924026; doi:10.3390/ijerph13060569)
Supplement: Supplementary file 1 [file ijerph-13-00569-s001.pdf]

Supplementary Materials: Interactions between Neighborhood Social Environment and Walkability to Explain Belgian Older Adults' Physical Activity and Sedentary Time

Veerle Van Holle, Jelle Van Cauwenberg, Ilse De Bourdeaudhuij, Benedicte Deforche, Nico Van de Weghe and Delfien Van Dyck

Table S1. Content and scoring of neighborhood social environment variables.

Variable Name [1] (Number of Items)	Content	Scoring
Talking to neighbors [2] (2)	How often do you say hello to a neighbor? How often do you stop and have a chat with a neighbor?	7-point scale (1 = never; 7 = (almost) daily)
Social interactions with neighbors [2] (3)	How often do you visit a neighbor, or receive a visit from a neighbor? How often do you go somewhere (e.g., to a shop; to a restaurant), together with a neighbor'; How often do you ask help/advice from or do you help/give advice to a neighbor yourself?	7-point scale (1 = never; 7 = (almost) daily)
Neighborhood [2] social trust and cohesion (5)	People in this neighborhood can be trusted; This is a close-knit neighborhood; People around here are willing to help their neighbors; People in this neighborhood generally don't get along with each other *(reverse coded)*; People in this neighborhood do not share the same values *(reverse coded)*	4-point Likert scale (1 = strongly disagree; 4 = strongly agree)
Neighborhood [2] social diversity (3)	Too many youngsters live in this neighborhood (reverse coded); Too many immigrants live in this neighborhood' *(reverse coded)*; Only older people live in this neighborhood *(reverse coded)*	5-point Likert scale (1 = strongly disagree; 5 = strongly agree)

[1] All variables were calculated by averaging the scores on the items included.; [2] The neighborhood was defined as the "environment around the home residence, within a walking distance of approximately one kilometer (the equivalent for a 10- to 15-min walk)' and neighbors were defined as 'non-family members above the age of 12".

Table S2. Associations between social environment factors and MVPA, and interactions with neighborhood walkability and income (single models).

Social Environment Factor	Main Effect Walkability	Main Effect Income (Ref. = Low)	Main Effect Soc. Env. Factor	Income × Walkability	Income × Soc Env. Factor	Walkability × Soc. Env. Factor	Walkability × Income × Soc. Env. Factor
	B ± SE	B ± SE	B ± SE	B ± SE	B ± SE	B ± SE	B ± SE
Talking to neighbors	**2.09 ± 0.72 ***	0.53 ± 0.69	0.313 ± 0.166	**−2.19 ± 0.59 ***	−0.370 ± 0.33	0.16 ±0.33	0.35 ± 0.65
Interactions neighbors	**3.26 ± 1.62 ***	2.20 ± 1.55	**0.317** ± 0.447	**−5.35 ± 2.18 ***	−0.74 ± 0.60	−0.51 ± 0.67	**1.48 ± 0.90 ¥**
Social trust & cohesion	**1.91 ± 0.78 ***	0.39 ± 0.74	0.748 ± 0.588	**−1.85 ± 1.07 ¥**	−0.44 ± 0.86	**−1.79 ± 0.82 ***	**2.51 ± 1.17 ***
Social diversity	**2.20 ± 0.73 ***	0.37 ± 0.71	0.323 ± 0.338	**−2.25 ± 0.99 ***	−0.53 ± 0.70	−0.53 ± 0.66	−0.92 ± 1.38

* $p < 0.05$; ¥ $p < 0.10$; The outcome variable (MVPA) was square root transformed; main effects, two-way and three-way interactions were calculated for each social environmental factor, adj. for number of valid accelerometer wearing days, number of accelerometer hours on valid days, gender, age, living situation, residential self-selection, car ownership and educational attainment.; Main and interaction terms in bold font ($p < 0.10$) were simultaneously included in a multivariable model (Table 2).

Table S3. Single models for main associations between social environment factors and walking/SB, and moderating effects of walkability.

Independent Variables	Walking Transport [1]	Walking Recreation [1]	Overall SB [2]	TV Viewing [3]
	B ± SE	B ± SE	B ± SE	B ± SE
**Main effects**				
Talking to neighbors	0.65 ± 0.23 *	080 ± 0.37 *	−0.00 ± 0.21	−0.10 ± 0.43
Social interactions among neighbors	−0.06 ± 0.31	0.52 ± 0.37	0.17 ± 0.21	−0.41 ± 0.18
Social trust & cohesion	0.49 ± 0.41	0.36 ± 0.48	0.12 ± 0.27	−0.00 ± 0.25
Neighborhood social diversity	−0.60 ± 0.67	1.14 ± 0.54 *	0.08 ± 0.31	−0.49 ± 0.29 ¥
**Interactions**				
Walkability × talking to neighbors	0.60 ± 0.45	0.12 ± 0.53	−0.56 ± 0.30 ¥	−0.36 ± 0.28
Walkability × social interactions among neighbors	−0.06 ± 0.62	0.58 ± 0.73	−0.63 ± 0.41	−0.66 ± 0.36 ¥
Walkability × social trust & cohesion	−0.04 ± 0.81	0.70 ± 0.94	0.58 ± 0.53	−0.51 ± 0.50
Walkability × social diversity	2.07 ± 0.91 *	−0.99 ± 1.0	0.88 ± 0.59	−0.23 ± 0.55

B = regression coefficient; SE = standard error; * $p < 0.05$; ¥ $p < 0.10$; All outcome variables were square root transformed; [1] adjusted for gender, age, living situation, residential self-selection, motorized vehicle ownership and educational attainment; [2] adjusted for number of accelerometer wearing days, number of accelerometer wearing hours per valid day, MVPA, gender, age, living situation, residential self-selection, motorized vehicle ownership and educational attainment; [3] adjusted for leisure-time PA, gender, age, living situation, residential self-selection, motorized vehicle ownership and educational attainment.

© 2016 by the authors. Submitted for possible open access publication under the terms and conditions of the Creative Commons Attribution (CC-BY) license (http://creativecommons.org/licenses/by/4.0/).
